# By Any Other Name: Heterologous Replacement of the *Escherichia coli* RNase P Protein Subunit Has *In Vivo* Fitness Consequences

**DOI:** 10.1371/journal.pone.0032456

**Published:** 2012-03-20

**Authors:** Paula C. G. Turrini, Jasmine L. Loveland, Robert L. Dorit

**Affiliations:** Department of Biological Sciences, Smith College, Northampton, Massachusetts, United States of America; Keio University, Japan

## Abstract

Bacterial RNase P is an essential ribonucleoprotein composed of a catalytic RNA component (encoded by the *rnpB* gene) and an associated protein moiety (encoded by *rnpA*). We construct a system that allows for the deletion of the essential endogenous *rnpA* copy and for its simultaneous replacement by a heterologous version of the gene. Using growth rate as a proxy, we explore the effects on fitness of heterologous replacement by increasingly divergent versions of the RNase P protein. All of the heterologs tested complement the loss of the endogenous *rnpA* gene, suggesting that all existing bacterial versions of the *rnpA* sequence retain the elements required for functional interaction with the RNase P RNA. All replacements, however, exact a cost on organismal fitness, and particularly on the rate of growth acceleration, defined as the time required to reach maximal growth rate. Our data suggest that the similarity of the heterolog to the endogenous version — whether defined at the sequence, structure or codon usage level — does not predict the fitness costs of the replacement. The common assumption that sequence similarity predicts functional similarity requires experimental confirmation and may prove to be an oversimplification.

## Introduction

Ribonuclease P (RNase P) is a ribonucleoprotein responsible for the processing of a number of RNA molecules critical to cell survival. It is central to the maturation of the 5′end of tRNAs, and also involved in the processing of other noncoding RNAs (2S, 4.5S, transfer-messenger-RNAs and small nucleolar RNAs) and in the recognition and cleavage of the bacterial riboswitches [1], [2], [3], [4], [5], [6], [7]. In the Eubacteria, RNase P is constituted by one RNA subunit encoded by the *rnpB* gene and one single protein subunit, encoded by the *rnpA* gene. Despite the demonstrated ability of the RNA moiety to perform catalysis *in vitro* under high ionic conditions, the protein subunit remains an essential component for the ribozyme activity *in vivo* [8], [9].

Considerable effort has been directed towards an understanding of the role played by the protein subunit in the holoenzyme and in the catalytic reaction. The protein moiety is central to the overall functionality of the enzyme: it establishes interactions that significantly influence the conformational structure of the RNA subunit and hence affect both holoenzyme formation and as the assembly of the holoenzyme-substrate complex [5], [10], [11], [12], [13]. Interestingly, the *rnpA* protein may not be performing an identical function in all organisms: its influence on the holoenzyme complex appears to depend on the identity of the RNA subunit [14].

The relationship between RNase P protein structure, function and evolution remains elusive. Comparative studies suggest that the tertiary structures of bacterial RNase P protein show remarkable conservation despite significant divergence in primary sequence [15], [16], [17], [18], [19]. As a result, highly divergent protein subunits taken from phylogenetically distant sources can still form a functional holoenzyme *in vivo* with the RNase P RNA subunit of *Bacillus subtilis* [17], [20].

While complementation studies provide a first glimpse into the potential interchangeability of molecular subunits, we were particularly interested in the evolutionary landscape occupied by the different versions of the RNase P protein. In effect, we wish to examine not only the ability of heterologous RNase P proteins to complement the deletion of the endogenous *rnpA*, but also to explore the fitness consequences of that complementation. Here, using an *E. coli rnpA* knockout strain, we examine the consequences of such heterologous complementation on key growth rate parameters: the highest achievable growth rate (µmax) and the lag time required to reach that maximum growth rate, a measure of growth acceleration we term *time to inflection* (TTI). We demonstrate *in vivo* that *E. coli* RNase P protein can be replaced by a broad range of heterologous versions of the protein and investigate the quantitative fitness consequences of making cell survival dependent on the function of a chimeric RNase P ribonucleoprotein, composed of the wild-type *E. coli* RNA subunit and a heterologous protein moiety.

Fitness, by definition, represents the integration of myriad contributions to the survival and reproduction of a given genotype. The measurement of fitness is made more elusive by the fact that fitness is generally environment-dependent. Despite these caveats, the importance of growth rate as a measure of bacterial fitness has been underscored by many studies [21], [22]. In these studies, increases in growth rate (often summarized by increases in maximal growth rate, µmax) in experimentally-derived genotypes suggested improvements in bacterial performance, i.e. improved fitness in determined environments. In this study, we maintain both the genetic background and the experimental environment constant throughout. As a result, the fitness consequences observed in our experimental *E. coli* lineages are directly measurable and attributable to the differences between the sequence of the heterologous *rnpA* and that of the native *E. coli rnpA*.

## Results

Regardless of their sequence or structural distance from the endogenous *E. coli* RNase P protein, all of the heterologous *rnpA* genes we examined successfully complement the deletion of the endogenous *E. coli rnpA*. These heterologs, derived from groups dispersed throughout the Eubacterial phylogenetic tree, encompass a broad range of sequence divergences, differing at anywhere from 21.7 to 78.1% of amino acid residues from the sequence of the wild-type *E. coli rnpA* sequence. When divergences are further corrected to account for unseen substitutions, the sequences we tested exhibit pairwise divergences that range from 0.27 (*Proteus mirabilis rnpA*) to 1.72 (*S. oralis rnpA)* amino acid substitutions per site. We thus confirm and extend the finding that endogenous bacterial RNase P proteins can be complemented *in vivo* by diverse phylogenetic heterologs [20], supporting tertiary structure and function conservation as defining features of the evolution of bacterial *rnpA* proteins.

**Figure 1:**
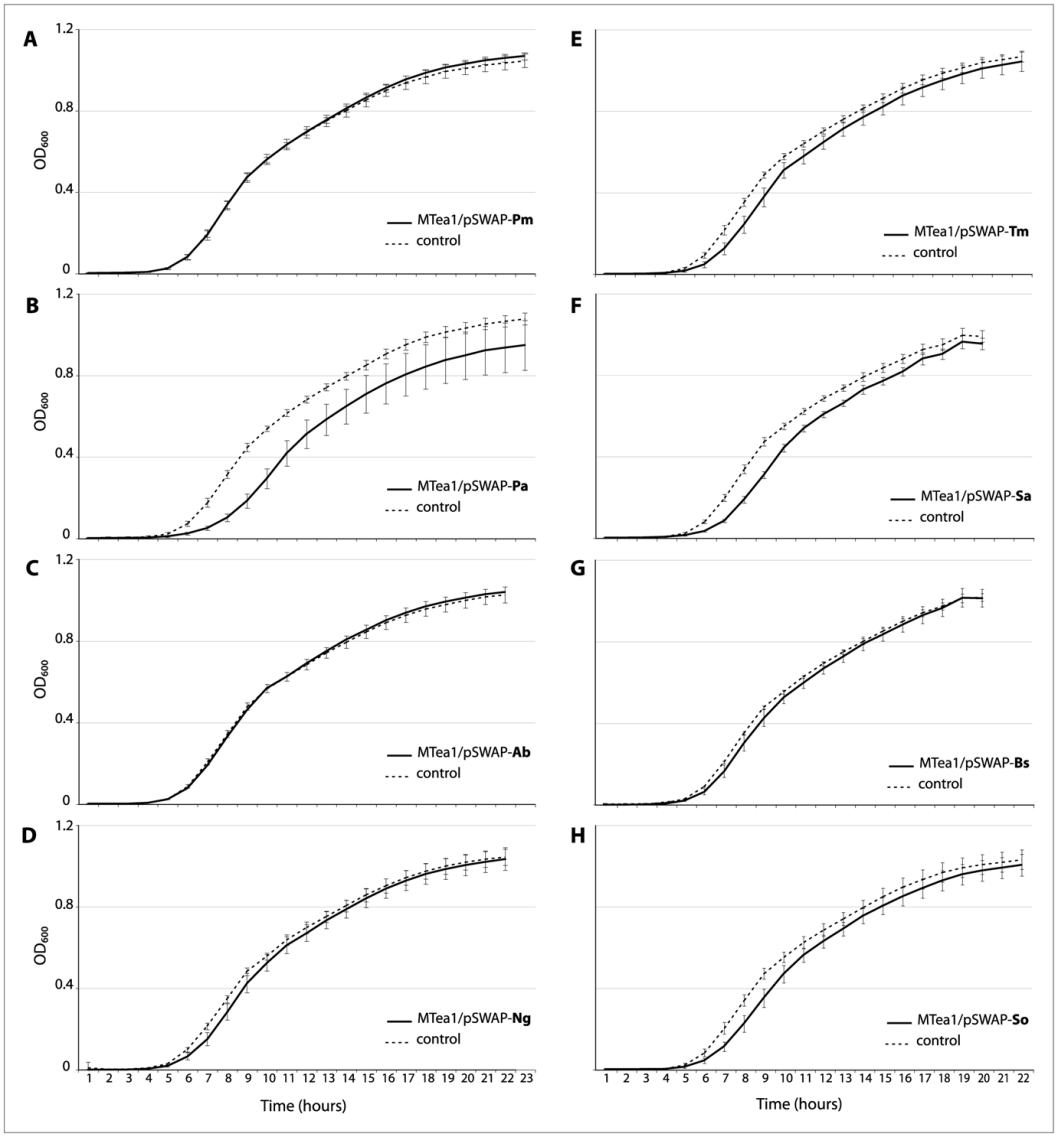
Growth curves of test lineages. Growth curves for each of the test lineages averaged over >20 replicates (solid lines) are compared to the control lineage (5 replicates) (dashed lines). Control lineage curves represent the growth of *E. coli* MTea1 expressing the wild-type *rnpA* in pSWAP (MTea1/pSWAP-Ec). The test lineages depicted harbor the following heterologous *rnpA*s in an MTea1 background: A) *P. mirabilis* (Pm), B) *P. aeruginosa* (Pa), C) *A. baumannii* (Ab), D) *N. gonorrhoeae* (Ng), E) *T. maritima* (Tm), F) *S. aureus* (Sa), G) *B. subtilis* (Bs), H) *S. oralis* (So).

We focused on the quantitative characterization of the correlation between sequence similarity and functional interchangeability of the *E. coli rnpA* protein, using growth in single-species liquid culture to examine the fitness of the test lineages. We define complete functional interchangeability as the capacity of an *rnpA* heterolog to rescue holoenzyme function—thus ensuring cell survival—with no subsequent reduction in fitness. These experiments thus explore the extent to which the effects of heterologous *rnpA* replacement map onto amino acid sequence similarity. Figure 1 suggests that the effects are, for the most part, subtle but of sufficient magnitude and reproducibility to be of clear evolutionary significance. In none of the panels shown in Figure 1 do the test lineages grow faster than the control lineage (which depends on a plasmid-encoded *E. coli rnpA* gene). The growth curves of lineages dependent on the *P. mirabilis*, *A. baumannii* and *B. subtilis rnpA* heterologs are virtually superimposed on the growth curve of the control lineage. In those cases where the curves are distinct, the test lineages appear to lag behind the control lineage; and in the case of *P. aeruginosa*, the asymptotic maximum density (“carrying capacity”) appears lower than it is for the test lineage.

**Figure 2:**
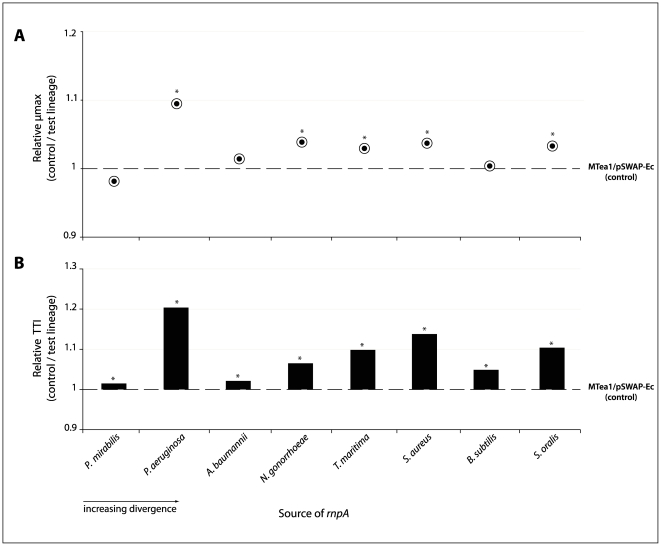
Effect of heterolog divergence on growth parameters of test lineages. A) Relative maximum specific growth rates (normalized to MTea1/pSWAP-Ec) for each test lineage. µmax is defined as the maximum growth rate in the Richards growth model [23]. B) Differences in the time to inflection of each test lineage relative to MTea1/pSWAP-Ec. The time to inflection (TTI) is defined as the length of time required to reach µmax. Values for the control lineage are arbitrarily set to 1. Asterisks indicate p values <0.001.

In order to explore the growth of our test lineages in more rigorous detail, the curves were fitted to a model of logistic growth [23], [24], from which we extract two key parameters, µmax and TTI, which act as proxies for fitness. These two parameters capture the dynamics of lineage growth more accurately than does µmax alone. Our approach reveals that all of the test lineages – *E. coli* hosts relying solely on a plasmid-encoded heterologous *rnpA* for survival – had either equivalent or higher maximum growth rates (µmax) under our growth conditions than did the control lineage. The test lineages containing *rnpA* from *P. aeruginosa*, *N. gonorrhoeae*, *S. oralis*, *S. aureus* and *T. maritima* exhibited significantly higher µmax rates compared to the reference MTea1/pSWAP-Ec strain. No significant differences were found between the control lineage and test lineages expressing *P. mirabilis*, *A. baumannii* or *B. subtilis rnpA* (Figure 2A; Table 1).

**Table 1:** Average growth parameters (µmax and TTI) from growth curves.

*E. coli* lineages	Average µmax (h^−1^)	Average TTI (hrs)	Relative µmax^a^	Relative TTI^b^
	± S.D.	± S.D.		
*Experiment 1*				
MTea1/pSWAP-Pm	0.661±0.010	7.65±0.16	0.982	1.015
MTea1/pSWAP-Ec	0.674±0.035	7.54±0.09		
*Experiment 2*				
MTea1/pSWAP-Pa	0.712±0.014	9.51±0.24	1.095	1.204
MTea1/pSWAP-Ec	0.652±0.025	7.95±0.41		
*Experiment 3*				
MTea1/pSWAP-Ab	0.687±0.019	7.62±0.26	1.014	1.021
MTea1/pSWAP-Ec	0.678±0.012	7.46±0.06		
*Experiment 4*				
MTea1/pSWAP-Ng	0.694±0.022	6.91±0.26	1.039	1.065
MTea1/pSWAP-Ec	0.668±0.014	6.49±0.08		
*Experiment 5*				
MTea1/pSWAP-Bs	0.711±0.025	7.82±0.21	1.004	1.049
MTea1/pSWAP-Ec	0.709±0.007	7.46±0.05		
*Experiment 6*				
MTea1/pSWAP-So	0.704±0.030	8.32±0.21	1.033	1.104
MTea1/pSWAP-Ec	0.681±0.017	7.53±0.13		
*Experiment 7*				
MTea1/pSWAP-Sa	0.747±0.019	8.51±0.11	1.037	1.138
MTea1/pSWAP-Ec	0.720±0.020	7.48±0.08		
*Experiment 8*				
MTea1/pSWAP-Tm	0.675±0.020	8.23±0.24	1.030	1.099
MTea1/pSWAP-Ec	0.655±0.010	7.49±0.14		

a Relative µmax means the ratio between the average µmax of each test lineage and the average µmax of the control lineage MTea1/pSWAP-Ec.

b Relative TTI means the ratio between the average TTI of each test lineage and the average TTI of the control lineage MTea1/pSWAP-Ec.

In contrast, the inflection points (TTI) of the growth curves for all test lineages, regardless of their maximum growth rates, were significantly above that of the reference lineage, meaning that they always reached their respective maximum growth rates later than did the control lineage MTea1/pSWAP-Ec (Figure 2B; Table 1). Differences in time to inflection reflect the ability of a given strain to translate available resources into growth. For bacteria, this parameter is seen as a critical component of fitness. We were mindful, however, of the possibility that the differences we were seeing in TTI simply reflected differences in the concentration of cells at the start of the experiment (Paw Dalgaard, pers. comm.). We thus examined the correlation between the cell density in our initial inocula and TTI values: none was found (Table S1; Figure S1).

**Figure 3:**
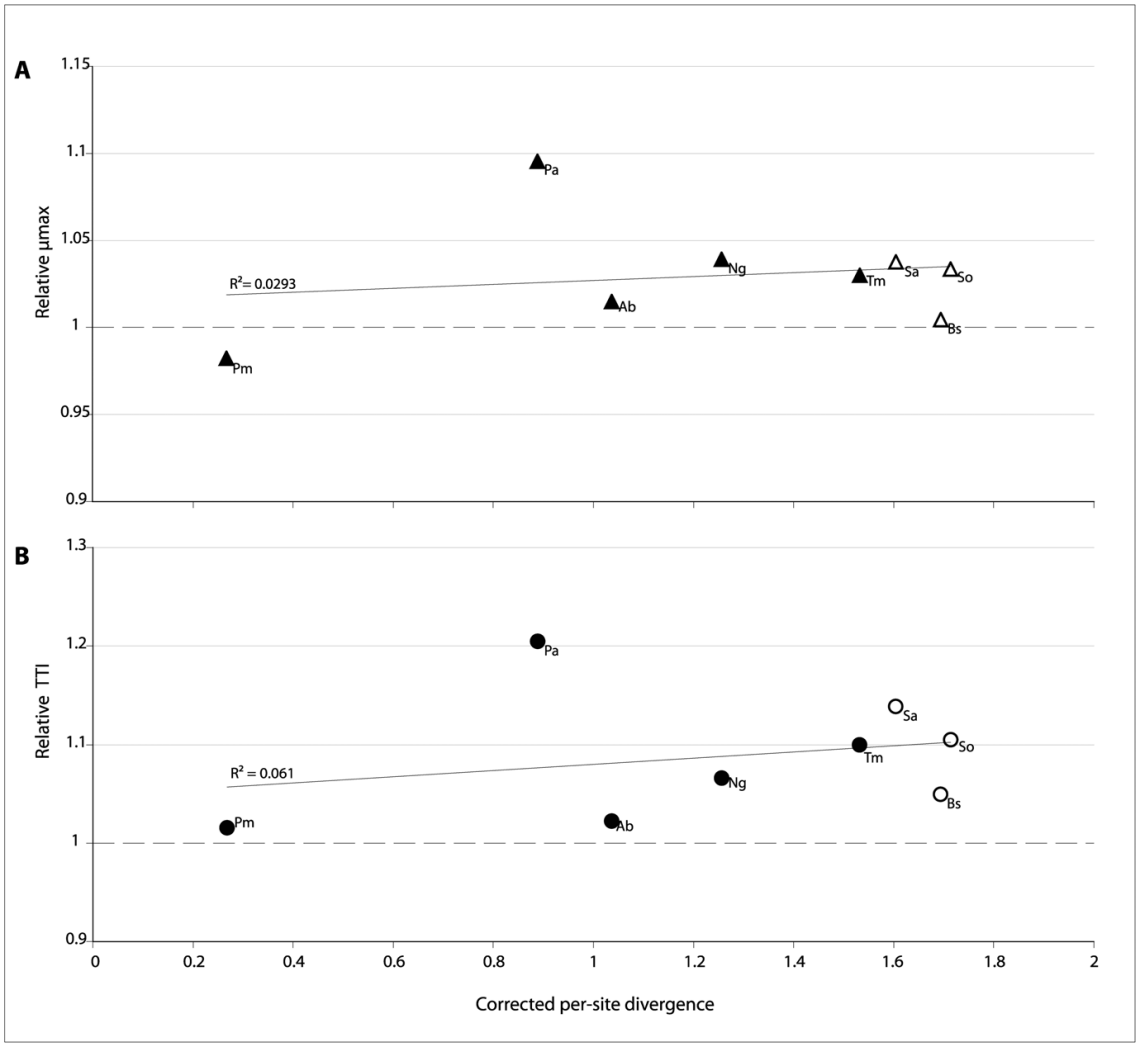
Correlation between *rnpA* divergence and growth parameters in test lineages. Panels A) and B) show the relationship between the previously defined growth parameters (µmax and TTI, respectively) and the corrected per-site divergence (number of amino-acid substitutions per amino-acid site using JTT substitution model as implemented in MEGA5.0 [51]). The dotted line represents growth parameter values for the *E. coli* control lineage, arbitrarily set to 1. Symbols are labeled to indicate the source organism of the *rnpA* heterolog present in pSWAP: Ab, *A. baumannii*; Bs, *B. subtilis*; Ec, *E. coli*; Pa, *P. aeruginosa*; Pm, *P. mirabilis*; Ng, *N. gonorrhoeae*; Sa, *S. aureus*; So, *S. oralis*; Tm, *T. maritima*. Solid line represents best-fit linear regression.

The extent to which sequence similarity predicts functional interchangeability is explored in Figure 3. The *rnpA* heterologs used in this study were deliberately chosen to span a range of phylogenetic distances (from the closely related *P. mirabilis* to the very distant *S. oralis*), as well as to include representatives associated with the two broad structural classes of RNase P RNA (“Type-A” and “Type-B”) [25], [26], [27]. There is virtually no correlation between the extent of sequence similarity and the effects on fitness (measured as µmax and TTI) of the heterologous replacement. The simple prediction that greater sequence divergence between the heterolog and the endogenous *E. coli rnpA* will result in larger fitness costs to the replacement is not borne out. It also appears that heterologs associated with the RNase P RNA class to which *E. coli* belongs (“Type-A”, shown as open symbols in Fig. 3) do not result in lower fitness costs when compared with their “Type-B” associated counterparts.

The functional equivalency of *rnpA* heterologs can also not be predicted using structural similarity. We complemented *E. coli* with three *rnpA* proteins with resolved three-dimensional structures: *T. maritima*, *S. aureus* and *B. subtilis* [17], [18], [19] whose *rnpA* products exhibit a high degree of structural conservation, similarity in their arrangement of α-helices and β-sheets, and comparable distributions of surface charges and core hydrophobic residues [17]. Despite being virtually indistinguishable at the structural level, complementation of the *rnpA* knockout strain (MTea1) with the *rnpA* proteins from these three organisms, gave rise to three different outcomes. Replacement with the *B. subtilis* protein had no effect on µmax and led to a small increase in TTI. In contrast, replacement with the *S. aureus rnpA* protein (although associated with type-B RNA) has little negative impact on either µmax or TTI.

**Figure 4:**
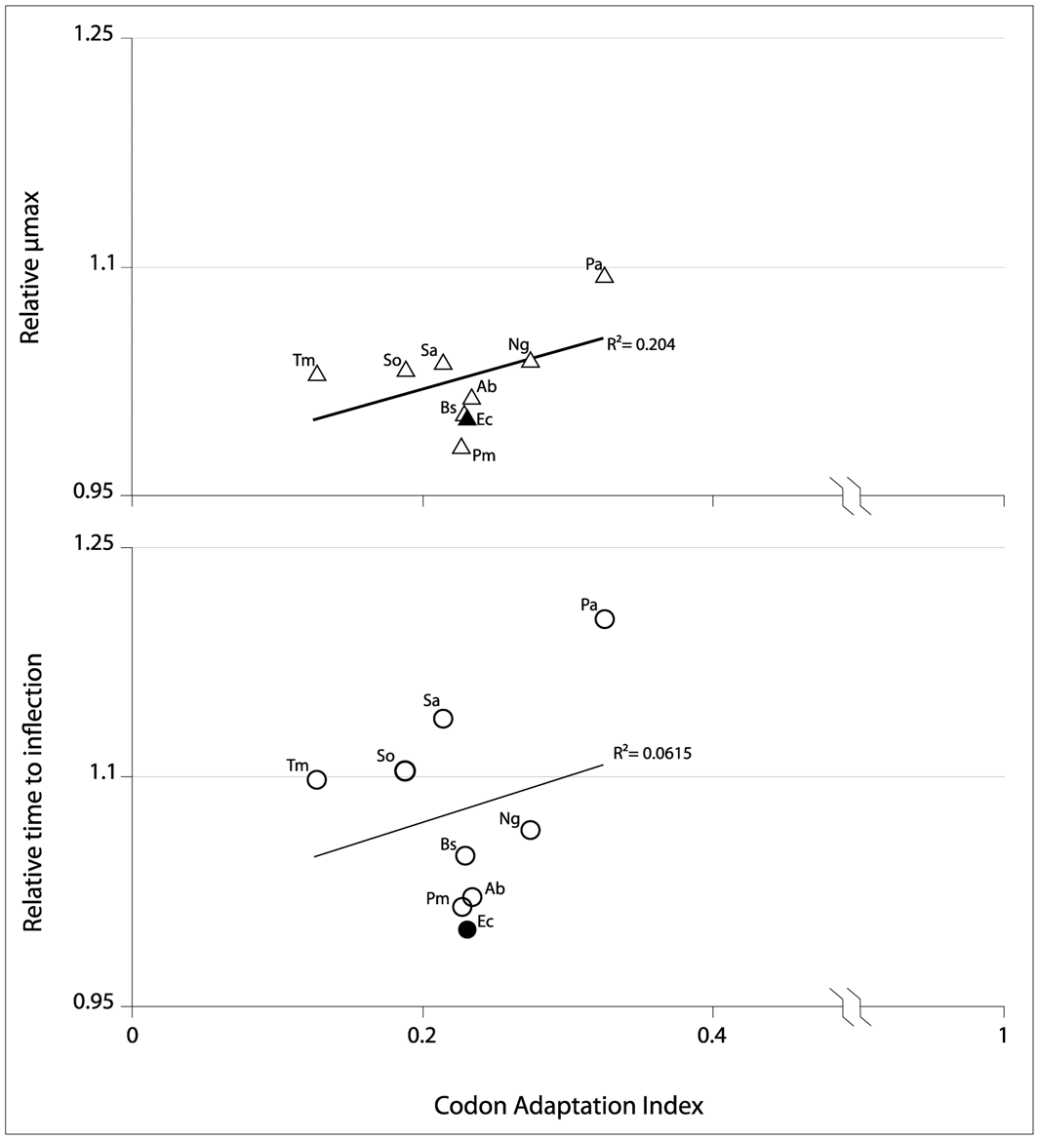
Correlation between growth parameters of test lineages and the Codon Adaptation Index (CAI). Solid symbols represent the values for *E. coli*; open symbols represent heterologous *rnpA* CAI and growth parameters of the test lineages, labeled as follows: Ab, *A. baumannii*; Bs, *B. subtilis*; Ec, *E. coli*; Pa, *P. aeruginosa*; Pm, *P. mirabilis*; Ng, *N. gonorrhoeae*; Sa, *S. aureus*; So, *S. oralis*; Tm, *T. maritima*.

Finally, we investigated the possibility that the fitness effects of heterologous replacement we describe could come about because of differences in codon usage of the heterologs (relative to the characteristic *E. coli* codon usage). Our measure of codon usage, the CAI, measures the extent to which the codon bias in the gene of interest (in this case *rnpA*) matches the bias in a previously identified reference set of high-expression genes [28]. The fact that the codon bias of the native *E. coli rnpA* (CAI∼0.25) is itself poorly matched to the overall codon bias of *E. coli* has been previously noted [29], [30], [31]. The CAIs of the tested heterologs do not differ dramatically, but nonetheless span the range from 0.127 to 0.325. However, as shown in Figure 4, there is no correlation between the growth parameters (µmax and TTI) and CAI.

## Discussion

As molecular evolutionists we assume, at least as a first approximation, that sequence similarity and functional interchangeability are correlated. Recently diverged orthologs generally share greater sequence similarity, and are thus less likely to have diverged in their function. Conversely, we do not necessarily expect that the deletion of an endogenous gene will be successfully complemented by a significantly divergent homolog. This study set out to explore the fitness consequences of replacing the endogenous protein component of the *E. coli* RNase P with increasingly divergent heterologs drawn from progressively more distant branches of the bacterial tree. We expected that phylogenetic relatedness and sequence similarity would correlate tightly with the fitness costs of the replacements. Surprisingly, complementing the knockout *rnpA E. coli* strain with increasingly divergent *rnpA* proteins did not reveal the expected association between sequence similarity and functional equivalence. We substituted the wild-type *rnpA* with heterologs that share only 11 (out of 120) fully conserved residues, and that otherwise exhibit between 21.7 and 78.1% uncorrected amino acid divergence relative to the endogenous *E. coli* sequence. Unexpectedly, reliance on increasingly divergent versions of *rnpA* to complement the otherwise lethal endogenous *rnpA* deletion does not necessarily result in increasing fitness costs to the *E. coli* host. Stated differently, the accumulating sequence divergence embodied by the heterologs in this study appears not to predict the fitness costs of heterologous complementation.

Our results indicate that the evolutionary history of *rnpA* has not led to a tight correlation between sequence similarity and functional interchangeability. This lack of correlation, however, does not imply that the species-specific evolutionary changes seen in the *rnpA* protein have no functional or fitness consequences. Indeed, our data reveal that, in every case, complementation of *E. coli* RNase P RNA with divergent *rnpA* proteins results in statistically significant fitness consequences. While the growth curves of the test lineages may, at first glance, appear similar to that of the control lineage, a more detailed analysis always reveals significant differences between test and control lineages for the two demographic parameters (µmax and TTI) used to analyze growth.

The consequences of heterologous replacement on µmax are initially surprising. They suggest that when the *E. coli* version of the RNase P protein — presumably coevolved to work in conjunction with the *E. coli* RNase P RNA moiety — is replaced with a heterolog, growth rate inevitably increases. Can this really mean that the endogenous version of *rnpA* confers the lowest fitness of all of the alternatives tested? We suggest instead that the biological significance of the increase in µmax seen in the lineages dependent on heterologous *rnpA* expression is best understood by recalling that µmax, while a key parameter of logistic growth, does not capture all components of fitness. In effect, µmax is a single parameter that captures the maximal growth rate attained under laboratory conditions (single species, excess carbon source, full oxygenation). Important as this parameter is, it would be premature to conclude that heterologous *rnpA* sequences result in fitter *E. coli* test lineages in all environments or under all growth conditions.

A closer look at our results suggests that the second parameter, TTI, may be at least as relevant to estimating the overall fitness of the complemented lineages. TTI, in effect, is a measure of acceleration, revealing the speed with which existing resources can be converted into cell growth. The fitness costs of replacement may be most apparent in this measure: all of the test lineage TTIs are significantly higher than that of the control lineage. The observed differences in TTI represent the impact of the heterologous complementation on the initial phases of the growth cycle: the lag and acceleration phases. While TTI is not commonly evoked in the analysis of growth curves, it has proven particularly useful for our comparative study. Because the growth kinetics are normalized to the behavior of the control strain containing the endogenous *E. coli rnpA* (albeit in a synthetic, plasmid-based location), small differences in TTI have a significant effect on overall fitness. In our system, acceleration (TTI) may be a more important indicator of overall fitness than is maximum speed (µmax). We reiterate that any experimental measurement of fitness can only capture a subset of fitness components, and that fitness measurements are always context and environment dependent. While the fitness advantages conferred by the endogenous *E. coli* RNase P protein in this study are captured primarily by the TTI parameter, the relative benefits of *E. coli rnpA* may well be more apparent under non-optimal growth conditions.

The heterologs chosen for this study were meant to encompass sequences located both near (*P. mirabilis rnpA*) and far (e.g. *B. subtilis rnpA*) in sequence space from the *E.coli rnpA*. Similarly, our candidates include RNase P proteins associated with the two previously described RNase P RNA structural classes (Type-A, including *E. coli* RNase P RNA and Type-B, including *B. subtilis* and *S. aureus*). Neither sequence similarity nor structural relatedness, however, predicted the fitness consequences of heterologous replacement (Fig. 2A and B, 3A and B).

Any attempt to correlate sequence similarity or difference (genotype) with function or fitness (phenotype) must be informed by our growing understanding of the mapping function that links these two fundamental levels. Work over the past decade has fleshed out the observation that multiple biomolecular sequences (RNA or protein) can converge on a single structure by revealing the presence of neutral networks [32], [33], [34]. These networks are trajectories that can traverse large distances in sequence space while remaining virtually immobile in structure/function space [35], [36], [37], [38], [39]. Our results underscore the multiple meanings of neutrality in evolutionary theory. Because all of the heterologs tested here appear capable of adopting the necessary three-dimensional shape to restore the *in vivo* function of RNase P, the *rnpA* sequences we investigate may be part of a neutral network. On the other hand, the reproducible fitness costs of these heterologous replacements suggest that they are not strictly neutral with respect to fitness, but are instead at a selective disadvantage relative to the strain harboring the endogenous *rnpA*. The *in vivo* function of RNase P depends on the close interaction between the RNA and protein moieties: we anticipate that such interactions will have significant impacts on the shape and extent of neutral networks.

We also explored the extent to which the difference between the codon bias of the endogenous *rnpA* and that of the heterologs might predict the fitness costs of replacement. Once again, there seems to be little correlation, with heterologs sharing the CAI of *E. coli* resulting in either very similar (e.g. *P. mirabilis*) or significantly different (e.g *S. aureus*) growth rates. This result is perhaps less surprising, since the endogenous *E. coli rnpA* shows little codon bias (CAI∼0.25). This low codon bias in *E. coli* has been implicated in the regulation of *rnpA* expression [29], [40].

The ability of the *E.coli* RNase P to retain function even when forced to rely on highly divergent protein subunits also reflects the versatile function of the RNase P protein. The *rnpA* protein subunit performs a number of different roles in the function of RNase P, and these roles may not be fully conserved from one bacterial lineage to the next. The protein moiety has been implicated in the stabilization of RNase P [8], [41], [42], in the stabilizing of the RNase P-substrate complex and in enhanced product release [9], [13]. Furthermore, while essential *in vivo*, the basic functions of the protein component are dispensable *in vitro*. This versatility of roles suggests that the association between the RNA and protein components of bacterial RNase P may not be stringently sequence-dependent. That said, we note that the sequence of the RNase P protein evolves in a lineage-specific manner, and exhibits clear evidence of both selective constraint and of coevolution between the RNA and protein components (Loveland, et al., in prep). Using this same experimental system, we are currently exploring the fitness consequences of heterologous replacement of the RNA moiety of RNase P.

The quantification of the fitness consequences incurred by replacing the endogenous *rnpA* with increasingly divergent heterologs reveals unexpectedly complex patterns: although µmax of the lineages harboring heterologous *rnpA* appear constantly higher, the time required to reach µmax, as measured by the inflection point of the growth curve, is extended in these lines. This study underscores the importance of going beyond the simple binary outcomes of most complementation studies. Even in cases where heterologs can successfully complement deletion of a crucial gene, the fitness costs of the heterolog replacement are significant. Our prediction that the fitness consequences of heterologous replacement would correlate tightly with sequence similarity is not borne out by our experiments. Instead, by comparing the overall fitness of genotypes that vary only in the source of their RNase P protein, we show that the genetic distance between *rnpA* heterologs does not predict the direction, intensity or character of the fitness costs imposed by the replacement. The gradual accretion of amino acid differences does result in fitness effects, but the correlation is far from obvious. The prevalent assumption that sequence and structural similarity predict functional similarity may be an oversimplification.

## Materials and Methods

### Bacterial strains and plasmids

Bacterial strains and plasmids used in this study are summarized in Table 2.

**Table 2:** Bacterial strains and plasmids used in this study.

Species or plasmids	Source/Reference	Genotype/Features
*Bacterial strains*		
*E. coli* BW25113/pKD46^a^	CGSC 7739/ [25]	*F- lacI+ rrnBT14 ΔlacZWJ16 hsdR514*
		*ΔaraBADAH33 ΔrhaBADLD78*
*E. coli* MTea1	This study	*F- lacI+ rrnBT14 ΔlacZWJ16 hsdR514*
		*ΔaraBADAH33 ΔrhaBADLD78 Δ(rnpA)::cat*
*Proteus mirabilis* b	Smith College Collection	
*Pseudomonas aeruginosa* b	ATCC 10145	
*Acinetobacter baumannii* b	ATCC 17978D	
*Neisseria gonorrhoeae* b	ATCC 700825D	
*Bacillus subtilis* b	Smith College Collection	
*Streptococcus oralis* b	ATCC 9811	
*Staphylococcus aureus* b	Smith College Collection	
*Thermotoga maritima* MSB8^b^	[23]	
*Salmonella typhimurium* TT23216^c^	John Roth	
*Plasmids*		
pCA24N^d^	JW 36815/ [24]	
pSAVE	This study	Gm^r^; Amp^r^; *rnpA* and GFP expression
pSWAP-Ec^e^	This study	Kan^r^; *E. coli rnpA* expression
pSWAP-Pm^e^	This study	Kan^r^; *P. mirabilis rnpA* expression
pSWAP-Pa^e^	This study	Kan^r^; *P. aeruginosa rnpA* expression
pSWAP-Ab^e^	This study	Kan^r^; *A. baumannii rnpA* expression
pSWAP-Ng^e^	This study	Kan^r^; *N. gonorrhoaea rnpA* expression
pSWAP-Bs^e^	This study	Kan^r^; *B. subtilis rnpA* expression
pSWAP-So^e^	This study	Kan^r^; *S. oralis rnpA* expression
pSWAP-Sa^e^	This study	Kan^r^; *S. aureus rnpA* expression
pSWAP-Tm^e^	This study	Kan^r^; *T. maritima rnpA* expression

a *E. coli* host for the *rnpA* knockout protocol; acquired from *E. coli* Genetic Stock Center, Yale, USA.

b used for amplification of the *rnpA* gene.

c template for the amplification of the antibiotic cassette for the gene inactivation protocol; provided by John Roth, University of California, Davis, USA.

d template for the *gfp* amplification.

e Heterologous complementation plasmids.

### Cell culture conditions

Cells were routinely grown in LB broth (Luria-Bertani, Miller, Difco dehydrated - BD Diagnostics Systems, Franklin Lakes, NJ, USA) prepared at 25 g/L or maintained for short periods of time in SOC liquid medium (New England Biolabs, MA, USA) containing (when appropriate) 50 µg/ml ampicillin, 25 µg/ml chloramphenicol, 30 µg/ml gentamycin or 50 µg/ml kanamycin. L-arabinose (Sigma-Aldrich, St. Louis, MO, USA) was used at 20 mM to induce expression of pKD46 plasmid genes. Isopropyl-B-D-thiogalactosidase (IPTG) was used at 0.1 mM to increase expression of *rnpA* and green fluorescent protein (GFP) genes in pSAVE and pSWAP-derivative plasmids. Cells containing pKD46 were grown at 30°C. Cells not containing or requiring elimination of pKD46 were grown at 37°C.

### PCR conditions

PCR amplification of target genes was achieved in a GeneAmp PCR System 9700 Thermal Cycler (Applied Biosystems, Carlsbad, CA, USA) with Phusion Hot Start polymerase (Finnzymes, Espoo, Finland), following manufacturers recommendations (1× HF buffer, 1.5 mM MgCl_2_, 0.2 mM each dNTP, and 0.01 U/µl polymerase), except for primers which were used at a final concentration of 1–2 µM for each in a total reaction volume of 25 µl or 50 µl. Cycling conditions were: 98°C for 3–10 min; followed by 35 cycles at 98°C for 15 s, 55–68°C for 15 s and 72°C for 90 s; and a final extension at 72°C for 5 min. The annealing temperature varied according to the set of primers used for each amplification. Initial denaturation varied according to the type of template used (cells, genomic DNA, plasmid or smaller DNA fragments). Colony PCR refers to insertion of bacterial colonies directly into the PCR master mix. PCR amplicons were purified using Qiagen purification columns (Qiagen, Valencia, CA, USA) or Microclean (The Gel Company, San Francisco, CA, USA). Oligonucleotides (primers) were synthesized by IDT DNA Technologies (Coralville, IA, USA) (Table S2). Sequencing reactions of plasmids and DNA fragments were performed using a Big Dye Terminator kit (Perkin-Elmer) and run on an ABI 3100 automated sequencer at the Center for Molecular Biology (Smith College, Northampton, MA). Amplification products were run into 0.8–1.2% agarose GPG/LE (American Bioanalytical, MA, USA) made with modified TAE buffer (40 mM Tris-acetate, pH 8.0, 0.1 mM EDTA) and visualized by staining with SYBR Safe (Invitrogen, Carlsbad, CA, USA) at a dilution of 1×10^4^. [43], [44]

### Electroporation conditions

For the preparation of electro-competent cells, bacterial cells were grown until cultures reached OD_600_ = 0.5. Fifty milliliters of cell culture were collected in a sterile conical tube, pelleted (4000×g for 15 min) and washed three times in ice-cold 10% glycerol sterilized solution. After draining the last wash, the pellet was resuspended with the 10% glycerol solution up to 500 µl and divided in 50 µL aliquots. Bacterial transformations were performed in a BioRad electroporator (Bio-Rad Laboratories, CA, USA) at 2.5 kV, 25 uF and 200 Ω. After transformation, cells were resuspended in 950 µl SOC and incubated in an orbital shaker, at 37°C. To spread all transformation cells onto a unique agar plate, cells were pelleted (4000×g for 10 minutes, at room temperature) and resuspended in 60–70 µl SOC.

### Chromosomal inactivation of *rnpA*

The *in vivo* heterolog replacement system used in our studies is a modification of one already in use in our laboratory (Loveland et al, in prep.). This system was developed to permit the deletion of any essential gene (in this case, the native *rnpA* gene) from the bacterial chromosome and allows its complementation by virtually any alternate version of the protein. The *rnpA* gene was deleted from the *E. coli* chromosome using the Red System [45], and this *rnpA* knockout lineage was denominated *E. coli* MTea1. Because *rnpA* is an essential gene *in vivo*, we developed a rescue plasmid named pSAVE, which is temporarily responsible for expressing *E. coli rnpA* following the deletion of the endogenous *E. coli rnpA* from the chromosome. In pSAVE, the *rnpA* gene is cotranscribed with GFP under a lacZ promoter and this entire region is flanked by Flp-recognition-target (FRT) sequences oriented in the same direction. Cells containing pSAVE plasmid are referred to as *E. coli* MTea1/pSAVE. Sequencing of the entire region surrounding the deletion confirms that the chromosomal copy of *rnpA* has been deleted and replaced by a chloramphenicol-resistance cassette.

### Heterologous complementation system

**Figure 5:**
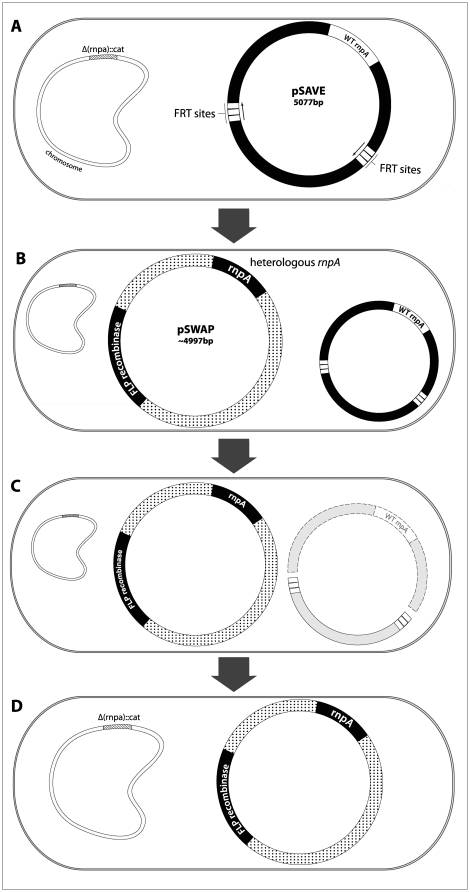
Complementation of *E. coli* with heterologous rnpA. A) The chromosomal copy of *rnpA* has been deleted from lineage MTea1 and replaced by an antibiotic marker (cat) (crosshatched). Cells survive this deletion by relying on the pSAVE plasmid to express the heterologous RNase P protein. pSAVE has an inducible copy of the native *E. coli rnpA* under the control of a lacZ promoter; this region is flanked by two Flp-recombinase targets (FRT); B) Lineages relying solely on the heterologous *rnpA* for survival were constructed by inserting a second plasmid, pSWAP, into MTea1. Besides the heterologous *rnpA*, pSWAP contains the Flp recombinase gene; C) Since FRT sites in pSAVE are oriented in the same direction, the expression of the pSWAP FLP recombinase promotes the deletion of the pSAVE fragment harboring the *E. coli rnpA* and encompassed by the FRT sites (dashed). The fragments of pSAVE are subsequently lost; D) The resulting *E. coli* lineage survives with the heterologous *rnpA* present in pSWAP, provided that the heterolog is capable of rescuing RNase P functionality.

In order to create *E. coli* strains that depended entirely on the activity of heterologous *rnpA*, we replaced the pSAVE plasmid with a different, incompatible plasmid, pSWAP. The pSWAP plasmid expresses a heterologous *rnpA* gene, as well as a Flp recombinase gene that targets the FRT sites flanking the *rnpA* region on pSAVE. Since FRT sites are oriented in the same direction [46], after transformation of pSWAP into MTEa1/pSAVE, Flp recombinase excises the *rnpA-gfp* region carried on the pSAVE plasmid: the *E. coli rnpA* and the remains of the pSAVE plasmid are subsequently degraded. The resulting strain can only survive if the orthologous *rnpA* being expressed in pSWAP is able to reconstitute RNase P function, since the unique functional *rnpA* available resides in pSWAP in the newly created lineage (Figure 5). Surviving strains are further screened for the loss of the endogenous *rnpA* by loss of fluorescence (present in pSAVE but not in pSWAP), antibiotic profile and diagnostic PCR and then selected for further fitness investigations. Complementing *E. coli* MTea1/pSAVE cells with a pSWAP plasmid harboring a nonfunctional *rnpA* sequence (pSWAP-null) displaced the plasmid pSAVE harboring the native *rnpA* and rendered all cells dead after transformation, confirming *rnpA* as an essential gene *in vivo*.

**Figure 6:**
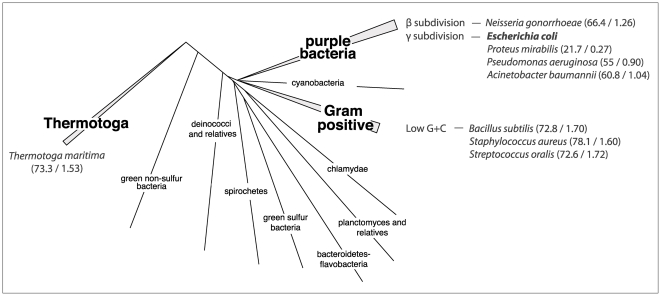
Phylogenetic range of *rnpA* heterologs investigated in this study. The phylogenetic position and range of sources of heterologous *rnpA* cloned into pSWAP for complementation and fitness studies are shown on a consensus eubacterial phylogenetic tree. The sampled branches are shown in boldface, and the species listed in italics alongside. *Escherichia coli* (bold) was used as control. The two numbers in the parentheses quantify the pairwise divergence between the heterologous and the *E. coli rnpA* sequences: the first number is the unadjusted pairwise amino-acid divergence across the entire length of the sequence (expressed as a percentage), the second the corrected per-site divergence (number of amino-acid substitutions per amino-acid site using JTT substitution model as implemented in MEGA5.0 [51]). Eubacterial phylogenetic tree adapted from [47].

We therefore constructed nine new *E. coli* lineages: eight ‘test’ lineages and one ‘control’ lineage. Test lineages harbor pSWAP containing a single divergent *rnpA* protein derived from different bacterial species and the control lineage harbors pSWAP containing the native *E. coli rnpA*. The nomeclature of newly created *E. coli* lineages reflects the origin of the *rnpA* gene expressed in the pSWAP plasmid (the control lineage MTea1/pSWAP-Ec is the *rnpA* knockout strain (MTea1) with pSWAP containing the wild-type *E.coli* version of *rnpA* (pSWAP-Ec)). We chose eight *rnpA* sequences from organisms across the Eubacterial phylogenetic tree [47] to represent the breadth of divergent sequences seen in Bacteria: four are from the Proteobacteria group - *Proteus mirabilis* (Pm), *Pseudomonas aeruginosa* (Pa), *Acinetobacter baumannii* (Ab) and *Neisseria gonorrhoaea* (Ng); three are from the Firmicutes group - *Bacillus subtilis* (Bs), *Streptococcus oralis* (So), and *Staphylococcus aureus* (Sa); and one is from the Thermotogae group - *Thermotoga maritima* (Tm) (Figure 6).

### Growth curve assays

We characterized the impact of heterologous substitution of *rnpA* using growth rate as a proxy for fitness. We compared the growth performance of each new test lineage relative to the native *rnpA*-containing control lineage – MTea1/pSWAP-Ec. We conducted a total of eight separate growth curve assays. Due to potential variation that could influence the growth of the cells in each experiment (media preparation, microplate positioning in the shaker, small differences in humidity and temperature in the incubator), each microplate represents one full experiment. Each growth curve assay consists of incubation of 23 clonal colonies of one test lineage and 5 clonal colonies of the control lineage MTea1/pSWAP-Ec, in triplicate. By using 23 clones of the test lineage, we intended to capture the possible variability of individual cells coping differently with the absence of the original *rnpA* protein in the RNase P holoenzyme. Assays were incubated at 37°C with orbital shaking (125 rpm) and optical densities (OD_600_) were recorded every full hour for 21–23 hours using SpectraMax M5 spectrophotometer (Molecular Devices, Sunnyvale, CA, USA) with a wavelength filter of 600 nm. Growth curves data were plotted as the averaged absorbance measurements at every time point (Figure 1).

### Fitness assessment

We modeled every growth curve generated. We used the Richards model [23] for curve fitting and extraction of the maximum growth rate (µmax) and time to inflection (TTI) parameters. The Richards model has been shown to accurately estimate maximum growth rate using absorbance data when the *m* parameter is fixed [24], using the following four-parameter equation:
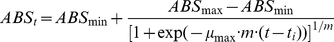
where ABS_t_ is absorbance at time *t*; ABS_min_ and ABS_max_ correspond to the asymptotic minimum and maximum, respectively; µmax is the maximum specific growth rate (h^−1^); t_i_ is the time at inflection point (TTI) and the fixed parameter *m* (empirically fixed at 0.5) describes the growth dampening. DataFit Software 9.0 (Oakdale Engineering, USA) was used to apply the Richards function to raw data. The mean of maximum growth rates (µmax) and time to inflection (TTI) of each test lineage were plotted relative to values of the MTea1/pSWAP-Ec control lineage grown in the same experimental microplate.

### Bioinformatic analysis

Sequence similarity was calculated comparing the native and the divergent *rnpA* proteins. The *rnpA* protein sequences were aligned using Seaview 4.1 [48] driving the multiple sequence alignment algorithm MUSCLE [49]. The resulting protein alignment was converted to a MEGA file format and the pairwise evolutionary distances were calculated using JTT matrix model of amino acid substitution [50] at uniform rates through MEGA5 [51].

### Codon Usage

We used the Codon of Adaptation Index (CAI) [28] to analyze the influence of codon usage in the expression of *rnpA* from different organisms being expressed in an *E. coli* host. CAI was calculated using CodonW [52] through MobylePasteur portal (http://mobyle.pasteur.fr), using the *E. coli* reference set of optimal codon usage.

#### Statistics

Statistical analyses were performed using STATA/SE 10.0 (StataCorp LP, TX, USA).

## Supporting Information

Figure S1 **Correlation between initial density (OD_600_) and growth parameters.** A) Correlation between initial density and the average µmax obtained from each growth curve experiment. B) Correlation between initial density and the average TTI obtained for each growth curve experiment. Points are labeled with the organism source of *rnpA* present in pSWAP: Ab, *A. baumannii*; Bs, *B. subtilis*; Ec, *E. coli*; Pa, *P. aeruginosa*; Pm, *P. mirabilis*; Ng, *N. gonorrhoeae*; Sa, *S. aureus*; So, *S. oralis*; Tm, *T. maritima*. Solid lines represent best fit linear regression.
(TIF)

Table S1 **Average optical density of the first inocula of test and control lineages in each growth curve experiment.**
(DOC)

Table S2 **PCR oligonucleotides used in this study.**
(DOC)
