# Supplementary figures and images for: By Any Other Name: Heterologous Replacement of the Escherichia coli RNase P Protein Subunit Has In Vivo Fitness Consequences
Source: PLoS One. 2012 Mar 20;7(3):e32456. doi: 10.1371/journal.pone.0032456 (PMC3308948; doi:10.1371/journal.pone.0032456)

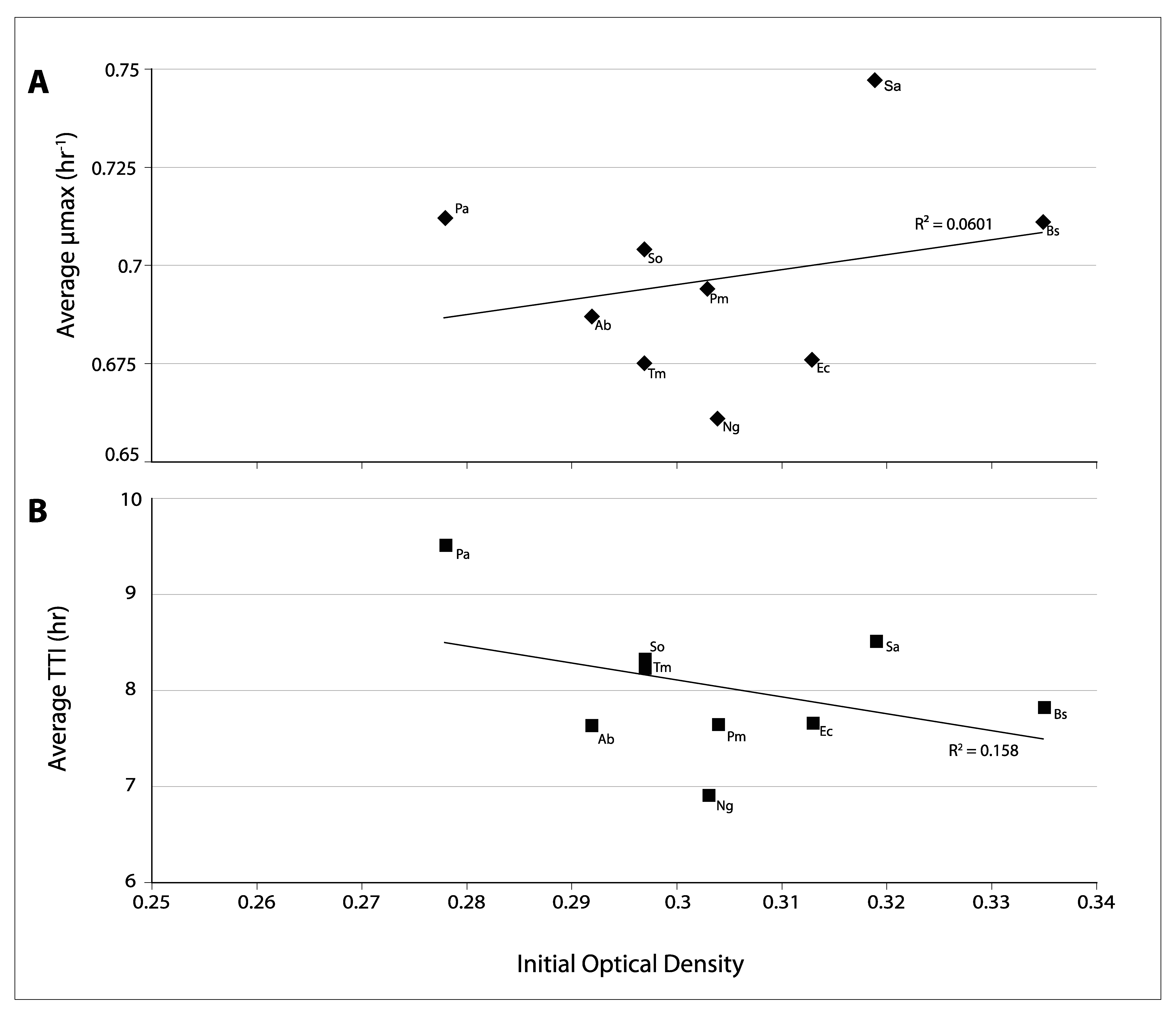

Supplement: Figure S1 — Correlation between initial density (OD600) and growth parameters. A) Correlation between initial density and the average µmax obtained from each growth curve experiment. B) Correlation between initial density and the average TTI obtained for each growth curve experiment. Points are labeled with the organism source of rnpA present in pSWAP: Ab, A. baumannii; Bs, B. subtilis; Ec, E. coli; Pa, P. aeruginosa; Pm, P. mirabilis; Ng, N. gonorrhoeae; Sa, S. aureus; So, S. oralis; Tm, T. maritima. Solid lines represent best fit linear regression. (TIF) [file pone.0032456.s001.tif]
