# Supplementary material for: By Any Other Name: Heterologous Replacement of the Escherichia coli RNase P Protein Subunit Has In Vivo Fitness Consequences
Source: PLoS One. 2012 Mar 20;7(3):e32456. doi: 10.1371/journal.pone.0032456 (PMC3308948; doi:10.1371/journal.pone.0032456)
Supplement: Table S1 — Average optical density of the first inocula of test and control lineages in each growth curve experiment. (DOC) [file pone.0032456.s002.doc]

Table S1. Average optical density of the first inocula of test and control lineages in each growth curve experiment.

| **Growth Curve Assays** | | **Average optical density of** |
| --- | --- | --- |
|  |  | **first inocula ± S.D.** |
| *Experiment 1* | |  |
|  | MTea1/pSWAP-Pm | 0.304 ± 0.019 |
|  | MTea1/pSWAP-Ec | 0.301 ± 0.0091 |
| *Experiment 2* | |  |
|  | MTea1/pSWAP-Pa | 0.278 ± 0.027 |
|  | MTea1/pSWAP-Ec | 0.306 ± 0.0281 |
| *Experiment 3* | |  |
|  | MTea1/pSWAP-Ab | 0.293 ± 0.011 |
|  | MTea1/pSWAP-Ec | 0.283 ± 0.0101 |
| *Experiment 4* | |  |
|  | MTea1/pSWAP-Ng | 0.303 ± 0.018 |
|  | MTea1/pSWAP-Ec | 0.308 ± 0.0151 |
| *Experiment 5* | |  |
|  | MTea1/pSWAP-Bs | 0.335 ± 0.015 |
|  | MTea1/pSWAP-Ec | 0.344 ± 0.0081 |
| *Experiment 6* | |  |
|  | MTea1/pSWAP-So | 0.297 ± 0.015 |
|  | MTea1/pSWAP-Ec | 0.305 ± 0.0081 |
| *Experiment 7* | |  |
|  | MTea1/pSWAP-Sa | 0.320 ± 0.014 |
|  | MTea1/pSWAP-Ec | 0.341 ± 0.0131 |
| *Experiment 8* | |  |
|  | MTea1/pSWAP-Tm | 0.297 ± 0.014 |
|  | MTea1/pSWAP-Ec | 0.295 ± 0.0141 |
