# Supplementary material for: By Any Other Name: Heterologous Replacement of the Escherichia coli RNase P Protein Subunit Has In Vivo Fitness Consequences
Source: PLoS One. 2012 Mar 20;7(3):e32456. doi: 10.1371/journal.pone.0032456 (PMC3308948; doi:10.1371/journal.pone.0032456)
Supplement: Table S2 — PCR oligonucleotides used in this study. (DOC) [file pone.0032456.s003.doc]

Table S2. PCR oligonucleotides used in this study.

| **Name of the primer** | **Sequence of the oligonucleotide^a^** |
| --- | --- |
| *For the construction of the antibiotic cassette* | |
| RDO0020 | CACCAAACACCCCCCAAAACCAAGCCACTG |
| RDO0021 | CACACAACCACACCACACCACACGGGGAGA |
| RDO0066 | GCCGCGCTCGTCTGACCGTTTCTAAGTAATAAAGCTAACCCCTGACACCAAACACCCCCC |
| RDO0067 | CCGAGTAGCGGACTAATCAGGCGTTGATAGACCCGAATGAGGGCTACACACAACCACACC |
| *For the confirmation of the rnpA knockout construction* | |
| RDO0070 | GGAAAGAGAATTGACTCCGGAGTG |
| RDO0071 | ATTTCGGGACGGGATCGTCACCACCA |
| *For the amplification of the insertion fragment* | |
| RDO0051 | TGTAATTTAGGTACC GCGGCCGCGTGAGTTAGCTCACTCATTAG |
| RDO0055 | CCCTGGGTCTAGAGACCTCCGTGTGAAATTGTTATCCG |
| RDO0068 | CCCTGGGTCTAGAATGGTTAAGCTCGCATTT |
| RDO0069 | AACCCGCCGAAGCGGGTTTTTACTTAAATCCCTGCAGGTCAGGACCCGCGAGC |
| RDO0056 | TAAATTACGGCCATTCAGGC GCGGCCGCAAAGCAAAAACCCGCCGAAGCGG |
| *For the amplification of heterologous rnpA genes* | |
| RDO0074 | CGATCGTCCTCTAGAATGAAGAAGCGAAATCG |
| RDO0075 | ATGTCTCGCCTGCAGGCTACTTTGACGAAGA |
| RDO0076 | CTCAGGACTTCTAGAATGGTGAGTCGGGAC |
| RDO0077 | ACAGATGCCCTGCAGGCTATGCATGAGTACC |
| RDO0084 | CCCACGCTCTAGAATGGAAAAAGCTTACCGAATT |
| RDO0085 | CCGCCACCTGCAGGTTACTTAATCTTTTTATTAAAAACTTTGGC |
| RDO0088 | CCAGGCTCTAGAATGGTTAAGCTCGCTTTTCCAAGGGAG |
| RDO0089 | CATGTTCCTGCAGGTCAGGATTTTTGAGCCAAGC |
| RDO0092 | CATTCTCTAGAATGACAGAGAGTTTCACCCGC |
| RDO0093 | TTACGCCTGCAGGTCATCCTTCTATCCTC |
| RDO0094 | CCTCCATCTAGAATGAAGAAAAGCTTTCGTG |
| RDO0095 | TTCTATCCTGCAGGCTAGTCAACTGTAGTTTCT |
| RDO0096 | CTTGATTCTAGAATGGACTACCGCTTCGGAAGGC |
| RDO0097 | GCCTTACCTGCAGGTCATACCTGTTTCCCG |
| RDO0100 | CCCATTCTAGAATGGCACTTTACAGTTTTAGC |
| RDO0101 | GCATCCCTGCAGGTTAATTCTGCGAGGTTG |

^a^ underscored nucleotides represent restriction enzyme sites embedded in the primer sequence
